# Characterization of intrathecal cerebrospinal fluid geometry and dynamics in cynomolgus monkeys (*macaca fascicularis*) by magnetic resonance imaging

**DOI:** 10.1371/journal.pone.0212239

**Published:** 2019-02-27

**Authors:** Mohammadreza Khani, Braden J. Lawrence, Lucas R. Sass, Christina P. Gibbs, Joshua J. Pluid, John N. Oshinski, Gregory R. Stewart, Jillynne R. Zeller, Bryn A. Martin

**Affiliations:** 1 Department of Biological Engineering, University of Idaho, Moscow, ID, United States of America; 2 School of Medicine, University of Washington, Seattle, WA, United States of America; 3 Department of Radiology, Emory University, Atlanta, GA, United States of America; 4 Axovant, New York, NY, United States of America; 5 Voyager Therapeutics, Cambridge, MA, United States of America; 6 Northern Biomedical Research, Spring Lake, MI, United States of America; Henry Ford Health System, UNITED STATES

## Abstract

Recent advancements have been made toward understanding the diagnostic and therapeutic potential of cerebrospinal fluid (CSF) and related hydrodynamics. Increased understanding of CSF dynamics may lead to improved detection of central nervous system (CNS) diseases and optimized delivery of CSF based CNS therapeutics, with many proposed therapeutics hoping to successfully treat or cure debilitating neurological conditions. Before significant strides can be made toward the research and development of interventions designed for human use, additional research must be carried out with representative subjects such as non-human primates (NHP). This study presents a geometric and hydrodynamic characterization of CSF in eight cynomolgus monkeys (*Macaca fascicularis***)** at baseline and two-week follow-up.

Results showed that CSF flow along the entire spine was laminar with a Reynolds number ranging up to 80 and average Womersley number ranging from 4.1–7.7. Maximum CSF flow rate occurred ~25 mm caudal to the foramen magnum. Peak CSF flow rate ranged from 0.3–0.6 ml/s at the C3-C4 level. Geometric analysis indicated that average intrathecal CSF volume below the foramen magnum was 7.4 ml. The average surface area of the spinal cord and dura was 44.7 and 66.7 cm^2^ respectively. Subarachnoid space cross-sectional area and hydraulic diameter ranged from 7–75 mm^2^ and 2–3.7 mm, respectively. Stroke volume had the greatest value of 0.14 ml at an axial location corresponding to C3-C4.

## Introduction

Cerebrospinal fluid (CSF) is a clear, colorless fluid with water-like mechanical properties that bathes the entire brain and spinal cord. CSF plays a role in the protection of neural structures, metabolic homeostasis of the central nervous system (CNS), autoregulation of cerebral blood flow, and immunological support for neural tissue. CSF moves freely in an oscillatory manner with approximately zero net flow, and in synchrony with cardiac-related intracranial pulsations and respiration [1–5]. Recent advances have been made toward understanding the diagnostic and therapeutic potential of CSF and related hydrodynamics. Increased understanding of CSF dynamics may lead to improved detection of CNS diseases, development of CSF system-based intrathecal drug delivery, and improved treatment of debilitating neurological conditions.

The importance of CSF dynamics has been investigated in several CNS conditions including syringomyelia [6] Alzheimer’s disease [7], Chiari malformation [8], and hydrocephalus [9]. Researchers have also applied computational fluid dynamics modeling approaches to understand how CSF dynamics related parameters could relate to CNS disease states and intrathecal drug delivery [10–14]. Before significant strides can be made toward the research and development of interventions designed for human consumption, additional research must be carried out with representative subjects such as non-human primates (NHP). However, relatively little information is known regarding CSF geometry and hydrodynamics in NHPs.

Studies have examined the possible role of CSF as a conduit for distribution of radiolabeled tracers [15] and therapeutic molecules to neuronal and glial cells of CNS tissues [16,17]. Intrathecal delivery of these molecules directly to the CNS tissue [2] is, in part, dependent on pulsation-dependent mixing of the spinal CSF dynamics. A solute injected into the CSF mixes [16], spreads throughout the CSF system, and is then taken up into the brain parenchyma via the perivascular (Virchow-Robbin) spaces [18,19]. Molecule injection to the CSF bypasses the blood-brain-barrier and allows delivery of many molecules that may not be possible through the systemic system [20,21]. The direct contact of CSF with neural tissue can enable delivery of small molecules to biologics including protein, cell-based, viral-mediated gene transfer, and gene therapies involving trophic factors to stimulate dying neurons [22,23]. These therapies have shown promise in animal studies [24,25] and safety in human clinical trials [26]. In addition, delivery in the CSF is a minimally invasive surgical intervention with a lower risk to the patient than other surgical interventions such as convection enhanced drug delivery and deep brain stimulation [25,27,28].

While intrathecal delivery of drugs or biologics to the CNS offers a promising treatment option, the dearth of knowledge has slowed therapeutic development and potentially confounded the analysis of therapeutic effectiveness. A common animal model used to test intrathecal therapeutics is the NHP, with one of the most common species being the cynomolgus monkey (*Macaca fascicularis*). Cynomolgus monkeys are a useful model for such studies since they are relatively compact compared to other NHP species, and share physiologic and cognitive similarities to humans. Despite being a frequently studied species, CSF hydrodynamic properties have not been studied or reported. At present, we do not know how NHP CSF hydrodynamics compare to humans or if they are consistent across animals and/or over time. The aim of the present study was to a) develop an MRI-based method to quantify intrathecal CSF dynamics in cynomolgus NHPs, b) use this method to quantify intrathecal CSF dynamics and geometry in a series of NHPs (N = 8), and c) measure the reliability of MRI-derived measures over a 2-week time interval.

## Materials and methods

### Ethics statement

This study was submitted to and approved by the local governing Institutional Animal Care and Use Committee at Northern Biomedical Research (IACUC approval #084-014A, Spring Lake, MI). This study did not unnecessarily duplicate previous experiments and alternatives to the use of live animals were considered. Procedures used in this study were designed with the consideration of the well-being of the animals.

### Animals

MRI measurements were obtained for eight (NHP 01–08) healthy five-year-old adult cynomolgus monkeys (Macaca fascicularis, origin Mauritius) from Charles River Research Models, Houston TX with a weight of 4.4 ± 1.2 kg (mean ± standard deviation). NHP 01 was male. All other NHPs were female (02–08). These animals were purpose-bred and experimentally naïve. Each NHP was scanned with an identical protocol at baseline and at follow-up after a 2-week time interval.

### MRI scan protocols

All MRI measurements were acquired at Northern Biomedical Research (Muskegon, Michigan, U.S.A.) on a Philips 3T scanner (Achieva, software V2.6.3.7, Best, The Netherlands). Prior to MRI scanning each NHP was prepared using standard procedures and precautions. NHPs were positioned in the scanner in the supine position without assistance from artificial respiration. During each scan, heart rate and respiration was monitored continuously with ~ 1 liter/minute of oxygen and 1–3% isoflurane anesthetic administered via endotracheal tube for sedation.

### Anatomic MRI scan protocol for CSF space geometry quantification

Total scan time to quantify CSF space geometry and flow (including NHP MRI preparation) for each NHP was ~1 hour after the protocols were in place. A stack of high-resolution axial T2-weighted MR images of the complete spinal subarachnoid space (SAS) geometry was acquired for each NHP using a VISTA (31 minutes) protocol (**Table 1**). The anatomical region scanned was ~30 cm in length, which included the intrathecal SAS below the lower brain stem extending caudally to the filum terminale. This comprised a total of ~720 images with 0.5 mm slice spacing, 1.0 mm slice thickness, and 0.375 mm isotropic in-plane resolution.

**Table 1:** Anatomic (T2-VISTA) and CSF flow (phase-contrast MRI) scan protocol parameters used for imaging cynomolgus monkeys.

Parameter	Anatomic (T2-VISTA)	CSF Flow (PC-MRI)
File size	941 MB	7 MB
Acquisition contrast	T2	Flow encoded
Acquisition type	3D	2D
Slice Thickness	1 mm	5 mm
Slice spacing	0.5 mm	N/A
Pixel bandwidth	481	192
Pulse Sequence	TSE	TFE
Transmit coil	Body	Body
Duration	31 minutes	200–240 seconds
Number of slices	660	N/A
Image matrix	864 x 864	224 x 224
In-plane resolution	0.375 mm	0.446 mm
Repetition time	2000 (ms)	11.226–12.704 (ms)
Echo Time	120 (ms)	6.749–8.226 (ms)
Cardiac phases	N/A	24
R-R interval	N/A	454–653 ms
Encoding direction	N/A	Thru-plane
Plane orientation	Sagittal	Axial
Trigger	N/A	Retrospective ECG
Velocity encoding	N/A	5 at FM and L4; 10 elsewhere

### Phase-contrast MRI scan protocol for CSF flow quantification

Thru-plane (head-foot, z-direction) CSF flow was measured by phase-contrast MRI (PC-MRI) images collected at six axial locations along the spine for each NHP. Axial locations were located at the foramen magnum (FM), C2-C3, C5-C6, T4-T5, T10-T11, and L3-L4 and required ~3 minutes scan time per location. Flow images were acquired with a retrospective ECG triggered sequence with 24 heart phases, 0.45 mm isotropic in plane resolution, and 5 mm slice thickness (**Table 1**). Slice location for each scan was oriented approximately perpendicular to the CSF flow direction with slice planes intersecting vertebral discs. More details on the PC-MRI protocol are given in Martin et al. [29].

### 3D image segmentation

**Fig 1:**
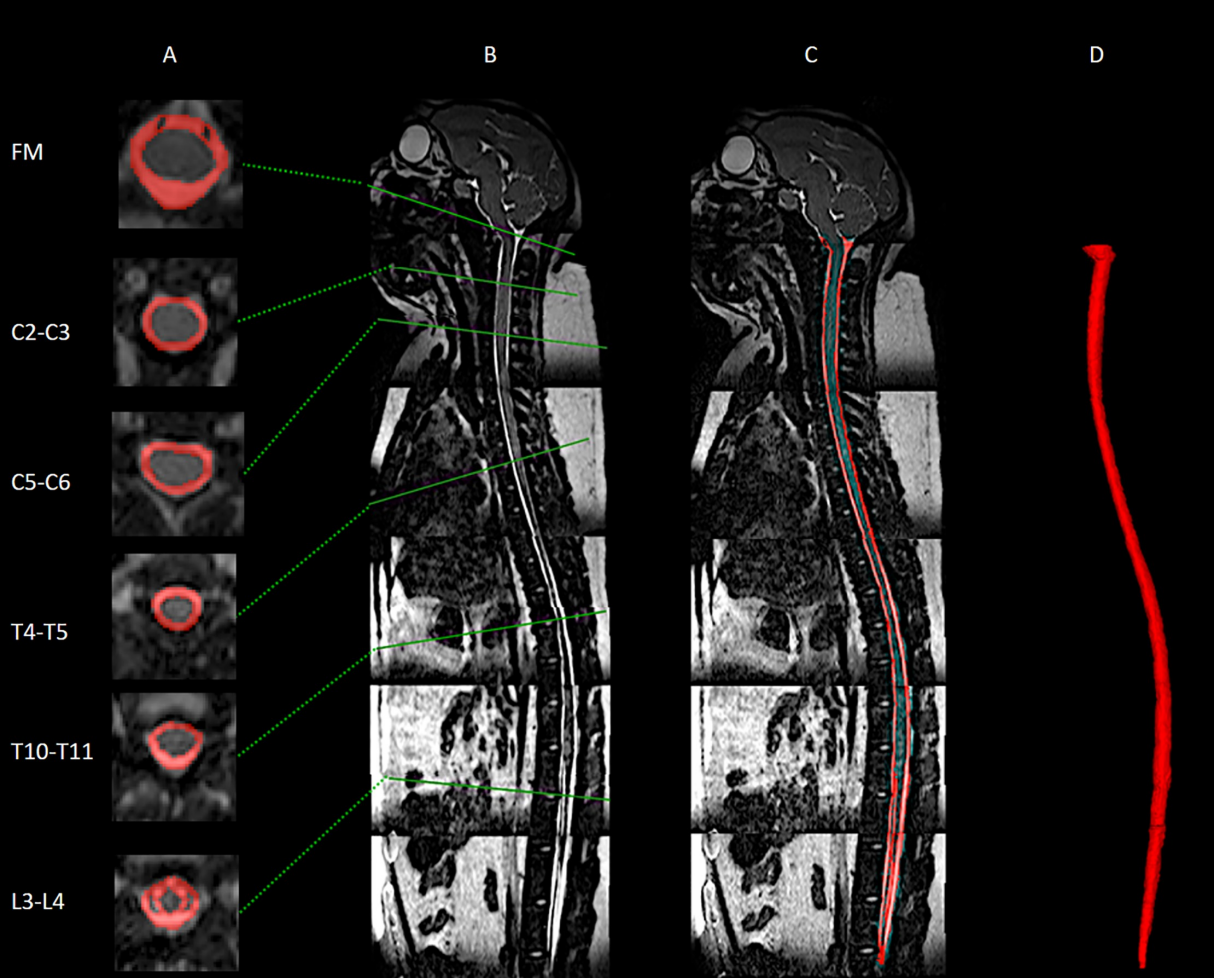
Manual segmentation of the spinal subarachnoid space using a T2-weighted MR image for a cynomolgus monkey analyzed in this study. (A) Visualization of SAS area manually selected around the spinal cord at multiple axial levels. (B) Mid-sagittal high-resolution T2-weighted MRI. (C) Sagittal visualization of segmented SAS around the spinal cord. (D) 3D visualization of entire SAS geometry. The same methods were applied to all MR images obtained for all NHPs.

The high-resolution T2-weighted anatomic MRI images were semi-automatically segmented using the free open-source ITK-snap software (Version 3.0.0, University of Pennsylvania, U.S.A.) [30], which provided semi-automatic segmentation using active contour methods, as well as manual delineation and image navigation (**Fig 1**). The manual segmentation tool was used most frequently with view of the three orthogonal planes. Detailed information on the segmentation procedure is provided by Martin et al. [31]. Once the segmentation was complete, the 3D model was exported in a .STL (Stereo Lithography) format for subsequent analysis as outlined below.

### CSF flow waveform and profile analysis

**Fig 2:**
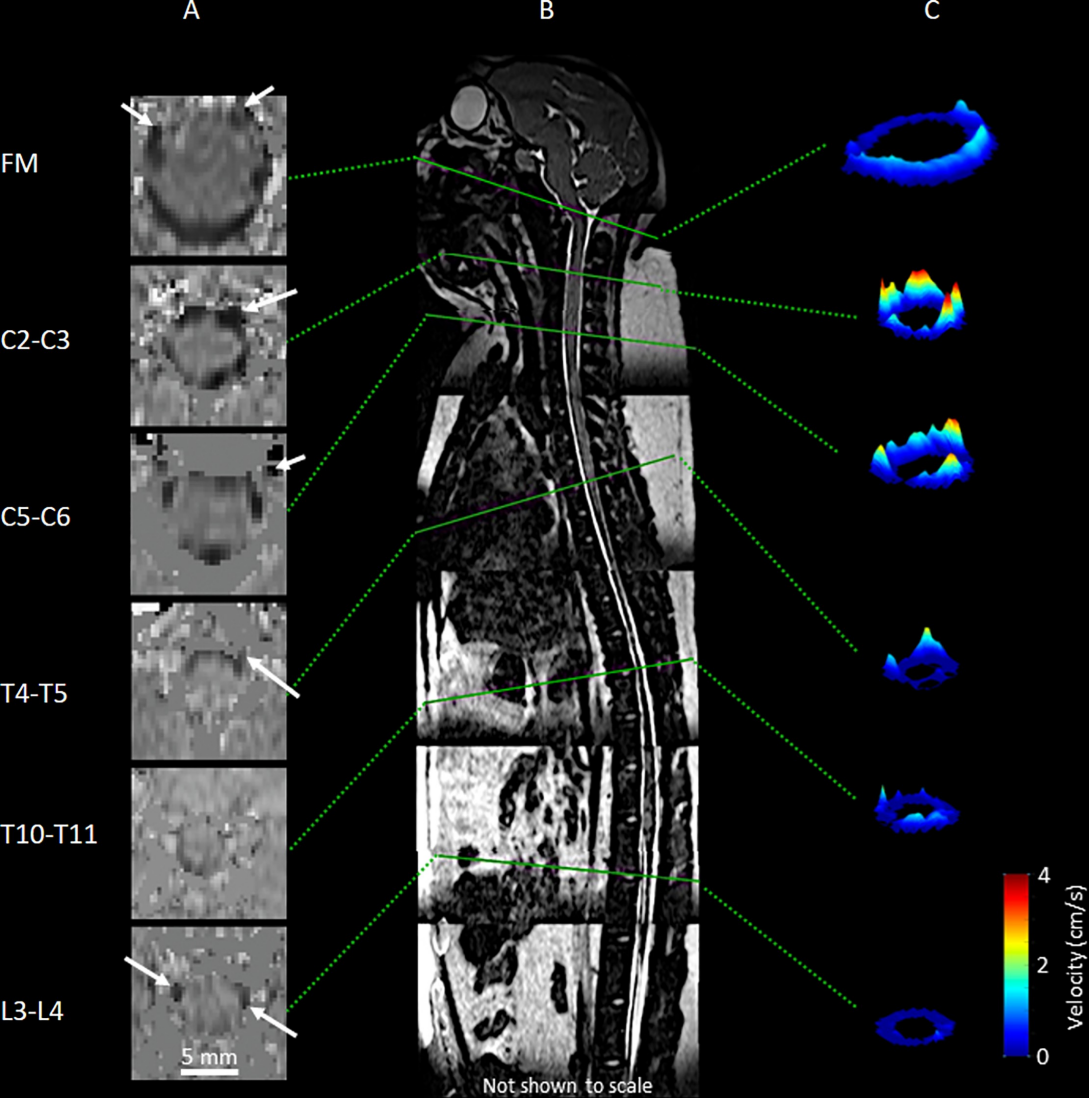
Axial PC-MRI and CSF velocity profiles at corresponding vertebral levels for a cynomolgus monkey in this study. (A) PC-MRI axial cross-sections for a given case at each respective vertebral level. (B) T2-weighted MR image of a cynomolgus monkey in the study with corresponding PC-MRI axial locations and slice orientation (solid green lines) at FM, C2-C3, C5-C6, T4-T5, T11-T12, and L3-L4. (C) 3D visualization of peak systolic CSF velocity profiles based on in vivo PC-MRI measurements at each vertebral level.

CSF flow was quantified at six axial locations along the spine (**Fig 2**) using GTFLOW software (64-bit, Version 2.2.10, Gyrotools, Zurich, Switzerland) by the following procedure previously described in [31]. PC-MRI and corresponding magnitude images were loaded into GTFLOW. A region of interest (ROI) was created within the area of CSF flow between the dura and spinal cord (**Fig 2A**). Individual pixel velocities within each ROI were exported to a .CSV (Comma-Separated Values) file for further analysis using MATLAB software (Ver. R2016a Mathworks Corp., Natick, MA). CSF flow waveform within the ROI, *Q*(*t*), was computed with *Q*(*t*) = ∑*A*_*pixel*_*V*_*pixel*_(*t*), where *A*_*pixel*_ is the area of one MRI pixel, *V*_*pixel*_(*t*) is the velocity for the corresponding pixel at any time, and *Q*(*t*) is the summation of the flow for each pixel within the ROI. The CSF flow waveform was offset to ensure zero net flow over the flow cycle since CSF flow in the spine has approximately zero net flow (oscillatory).

The following method was applied to generate a smooth spatial-temporal CSF flow distribution, *Q*(*z*,*t*), along the spine. The axial phase-contrast slice locations were not identical for each NHP due to differences of the exact vertebral levels across cases. Thus, the z-location of each slice was manually measured based on the distance of that slice caudal to the FM (**Table 2**). The six distinct flow rates were smoothed in a spatial-temporal fashion using MATLAB and a 2D “fit” function with the fit-type designated as “smoothing-spline”. Since heart rate variability was present between the PC-MRI scans, the CSF flow waveform timing was normalized to the average heart rate for all NHPs. An average spatial-temporal CSF waveform was determined. CSF pulse wave velocity, *PWV*, was computed based on the slope of the arrival time of peak CSF flow along the spine [32].

**Table 2:** Reference chart for vertebral disk location with respect to axial distance from the foramen magnum in cynomolgus monkeys.

Vertebral level	Mean ± std (mm)
'FM'	0.0 ± 0.0
'C1'	5.7 ±2 .4
'C2'	10.3 ± 2.6
'C3'	22.3 ± 3.9
'C4'	29.0 ± 3.6
'C5'	35.2 ± 3.8
'C6'	42.2 ± 3.4
'C7'	48.7 ± 3.8
'T1'	56.1 ± 4.2
'T2'	63.4 ± 4.4
'T3'	72.0 ± 4.2
'T4'	79.8 ± 4.2
'T5'	88.5 ± 4.5
'T6'	97.9 ± 4.9
'T7'	107.4 ± 4.5
'T8'	117.6 ± 4.8
'T9'	128.8 ± 4.5
'T10'	141.4 ± 5.4
'T11'	156.3 ± 5.0
'T12'	172.0 ± 5.1
'L1'	189.6 ± 5.4
'L2'	207.5 ± 5.8
'L3'	227.1 ± 6.8
'L4'	247.0 ± 7.0
'L5'	268.9 ± 6.2
'Sacrum'	289.4 ± 6.7
'coccyx'	301.0 ± 6.8

FM = Foramen magnum, C = Cervical, T = Thoracic, L = Lumbar

### Geometric and hydrodynamic parameter quantification

Several geometric and hydrodynamic parameters were calculated based on the 3D segmentation and flow analysis using our previously published methods [11]. Using the exported 3D .STL file (above), each of these parameters was calculated by a user-defined function (UDF) compiled in ANSYS FLUENT (ANSYS Academic Research, Release 19.1, Canonsburg, PA, USA) based on a computational mesh generated from ANSYS ICEM (ANSYS Academic Research, Release 19.1, Canonsburg, PA, USA). Details on the methods used to generate each parameter are as follows.

*The following parameters were computed based on overall spine geometry*: Total SAS surface area, *SA*_*sas*_, was calculated as the sum of surface area of spinal cord, *SA*_*c*_, and dura, *SA*_*d*_. Spinal cord nerve roots were not included in the surface area calculation of the cord since these small features were not possible to accurately visualize by MR imaging. Total volume of the SAS, *V*_*sas*_, was computed by subtracting the volume of the spinal cord, *V*_*c*_ from the volume of the dura, *V*_*d*_. An overall average, maximum, and minimum value was then computed across all NHPs. Total SAS length, *L*_*sas*_, from the FM to the SAS termination was quantified.

*The following parameters were determined for each 1 mm interval along the spine (z-location)*: Axial distribution of the SAS cross-sectional area, *A*_*sas*_(*z*), was based on cross-sectional area of the spinal cord at that location, *A*_*c*_(*z*), and dura, *A*_*d*_(*z*). Similarly, hydraulic diameter, *D*_*h*_(*z*) = 4*A*_*sas*_(*z*)/*P*_*sas*_(*z*), was determined based on the wetted perimeter, *P*_*sas*_(*z*), with the perimeter computed as the sum of the spinal cord, *P*_*c*_(*z*), and dura, *P*_*d*_(*z*), perimeters at each z-location. Axial distribution of CSF stroke volume was computed as *SV*(*z*) = ∫|*Q*(*z*,*t*)|*dt*, where |*Q*(*z*,*t*)| is the absolute value [33]. Peak systolic (toward feet) and diastolic (toward head) CSF flow rate was quantified as *Q*_*sys*_(*z*) and *Q*_*dia*_(*z*), and the CSF flow rate amplitude was given by *Q*_*a*_(*z*) = *Q*_*dia*_(*z*)−*Q*_*sys*_(*z*). Spatial mean thru-plane velocity at peak systole was computed as ${\bar{U}}_{sys}(z)=Q_{sys}(z)/A_{sas}(z)$ and at diastole as ${\bar{U}}_{dia}(z)=Q_{dia}(z)/A_{sas}(z)$. Reynolds number was computed as $Re(z)=\left({\bar{U}}_{sys}(z)\cdot D_{h}(z)\right)/v$, were *v* is the kinematic viscosity of CSF at body temperature, 0.693 mPa∙s [29]. Womersley number was computed as $\alpha (z)=\frac{D_{h}(z)}{2}\sqrt{\omega /v}$, where *ω* is the angular velocity (*ω* = 2*π*/*T*) of the volume flow waveform with *T* equal to the heart rate.

To allow parameter comparison across NHPs, each parameter’s axial distribution for each NHP was normalized to the average *L*_*sas*_ measured for all NHPs. After normalization, the mean axial distribution for each parameter was computed across all NHPs. The mean axial distribution was then used to obtain an average, maximum, and minimum parameter value along the spine based on all NHPs.

### Parameter reliability

Reliability was assessed by obtaining MRI measurements for each NHP at baseline and 2-week follow-up while ensuring identical methods during both collection intervals. To quantify measurement reliability, we performed a regression of baseline versus follow-up parameters computed at each axial location along the spine. All computations and plots were generated using MATLAB software (Ver. R2016a Mathworks Corp., Natick, MA).

## Results

Results were obtained for a total of eight NHPs at baseline and a 2-week follow-up time point (**Table 3**). Overall, the MRI protocol allowed quantification of all proposed geometric and hydrodynamic parameters. These parameters had a relatively similar axial distribution across all NHPs analyzed and were similar at follow-up for each NHP. CSF flow was laminar in all NHPs with the greatest degree of CSF motion observed in the cervical spine. Average results showed that maximum *Re* and *α* was 80 and 7.7, respectively. *A*_*sas*_ and *D*_*h*_ ranged from 7–75 mm^2^ and 2–3.7 mm, respectively. Maximum ${\bar{U}}_{sys}$ and ${\bar{U}}_{dia}$ was -2.7 to 1.6 cm/s and located in the cervical spine. *SV* ranged from 0.14 ml in the cervical spine to roughly 0 ml in the lower lumbar spine for all NHPs.

**Table 3:** Summary of geometric and hydrodynamic results. Mean values correspond to the average along the entire spine for all 16 NHPs (except for total surface area, volume, and *PWV*). Local maximum and minimum values are computed based the average for all 16 NHPs.

Parameter	Symbol	Unit	Average	Maximum	Minimum
Parameters computed at 1 mm intervals along the spine					
*Perimeter of spinal cord*	*P*_*c*_	*mm*	14.77	62.02	0.73
*Perimeter of dura*	*P*_*d*_	*mm*	22.06	38.63	9.54
*Perimeter of subarachnoid space*	*P*_*sas*_	*mm*	36.82	99.56	10.50
*Area of spinal cord*	*A*_*c*_	*mm*^*2*^	15.05	81.50	0.50
*Area of dura*	*A*_*d*_	*mm*^*2*^	39.59	137.45	7.61
*Area subarachnoid space*	*A*_*sas*_	*mm*^*2*^	24.54	75.10	6.98
*Hydraulic diameter*	*D*_*h*_	*mm*	2.68	3.73	2.02
*Reynolds number*	*Re*	*NA*	29.30	79.27	0.66
*Womersley number*	*α*	*NA*	5.50	7.67	4.15
*Mean velocity at peak systole*	${\bar{U}}_{sys}$	*cm/s*	-0.83	-0.02	-2.69
*Mean velocity at peak diastole*	${\bar{U}}_{dia}$	*cm/s*	0.58	1.59	0.02
*Flow rate at peak systole*	*Q*_*sys*_	*ml/s*	-0.20	0.00	-0.60
*Flow rate at peak diastole*	*Q*_*dia*_	*ml/s*	0.14	0.35	0.00
*Flow rate amplitude*	*Q*_*a*_	*ml/s*	0.33	0.94	0.00
*Stroke volume*	*SV*	*ml*	0.05	0.14	0.00
*Parameters computed based on the entire spine*					
*Surface area of spinal cord*	*SA*_*c*_	*cm*^*2*^	44.74	49.63	37.76
*Surface area of dura*	*SA*_*d*_	*cm*^*2*^	66.66	70.49	60.53
*Surface area of subarachnoid space*	*SA*_*sas*_	*cm*^*2*^	111.39	120.12	98.31
*Volume of spinal cord*	*V*_*c*_	*ml*	4.57	5.40	3.98
*Volume of Dura*	*V*_*d*_	*ml*	11.99	13.45	10.25
*Volume of subarachnoid space*	*V*_*sas*_	*ml*	7.41	8.47	6.24
*Length of subarachnoid space*	*L*_*sas*_	*mm*	301	306.98	295.80
*Pulse wave velocity*	*PWV*	*m/s*	1.13	3.45	0.73

### Geometric parameters

**Fig 3:**
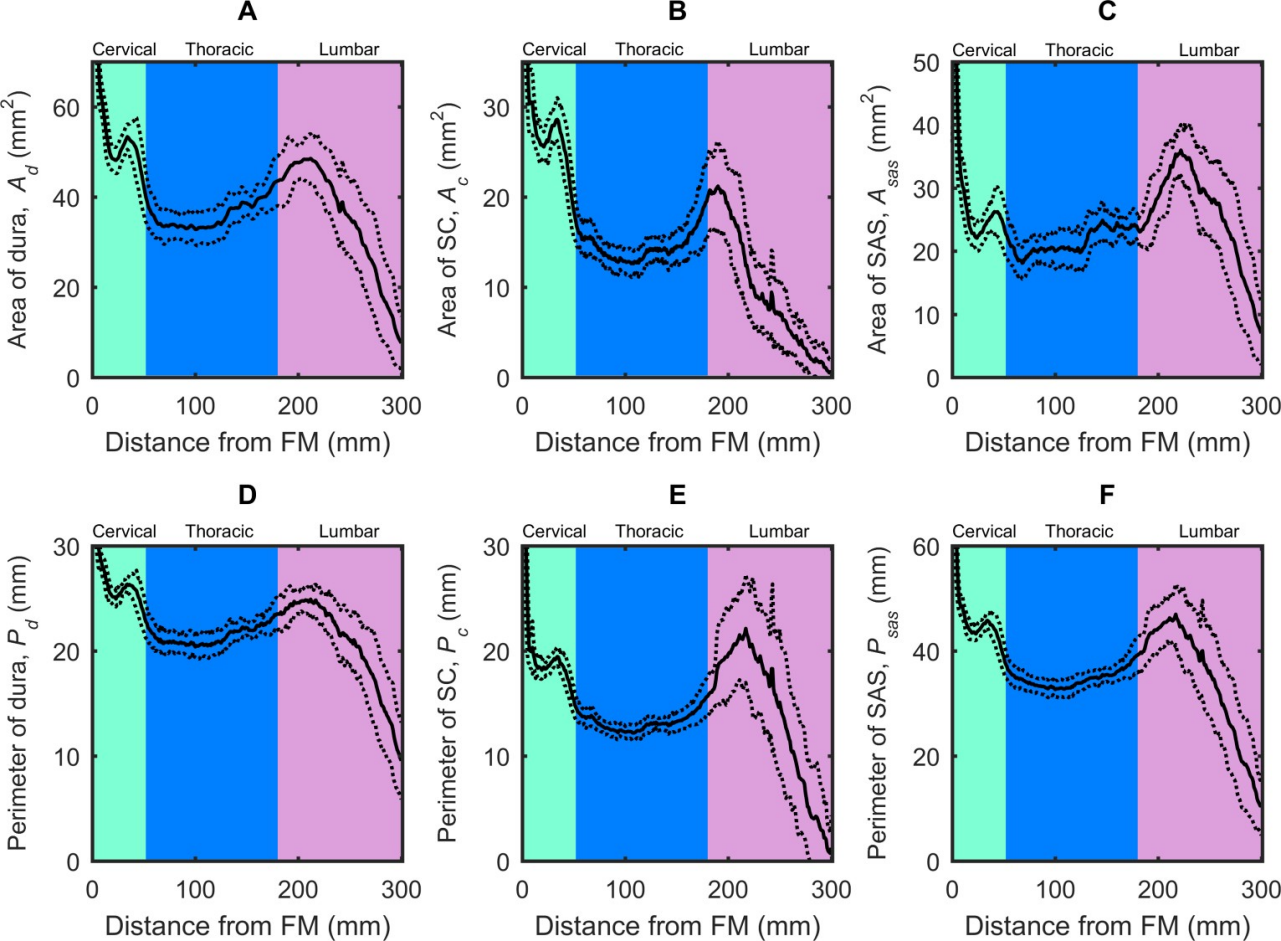
Geometric parameters distribution computed along the spine for cynomolgus monkeys. (A) Area of dura, (B) Area of spinal cord, (C) Area of subarachnoid space, (D) Perimeter of dura, (E) Perimeter of spinal cord, (F) Perimeter of subarachnoid space. Mean value for all 16 NHPs corresponds to the solid line. Dotted lines correspond to ± 1 standard deviation for all 16 NHPs analyzed.

Average *V*_*sas*_ for all NHPs was 7.41 ml. Average *SA*_*c*_ and *SA*_*d*_ was 44.74 ± 3.52 and 66.66 ± 3.11 cm^2^ respectively. *A*_*sas*_ and *D*_*h*_ decreased moving caudally down the spinal cord from the FM (**Fig 3**). The minimum value for *A*_*sas*_ and *D*_*h*_ was 7 mm^2^ and 2 mm, respectively (**Table 3**). These values occurred at ~70 mm caudal to the FM, a location approximately corresponding to T2-T3 (**Table 2**). Maximum difference in *A*_*sas*_ and *D*_*h*_ between NHPs at any axial location (omitting the FM) was ~30 mm^2^ and 4 mm, respectively.

### CSF flow waveforms

**Fig 4:**
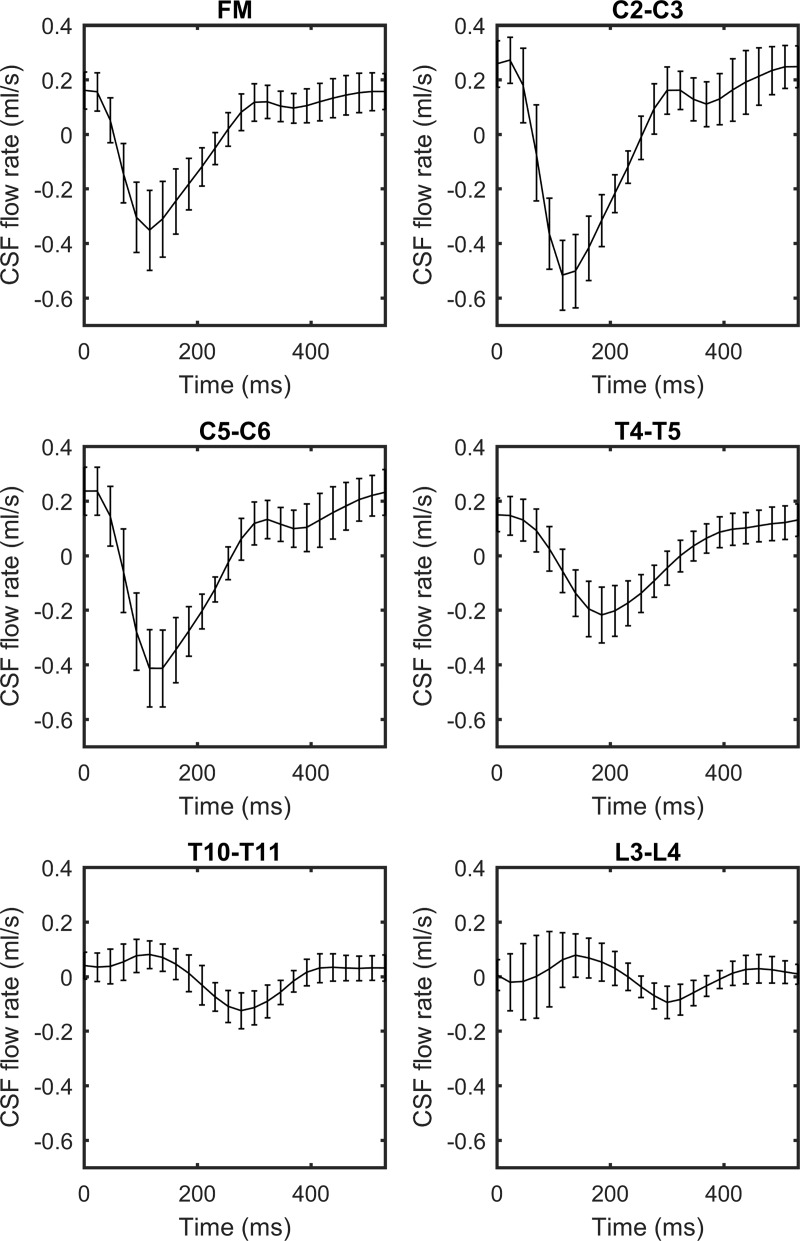
CSF flow waveforms for all 16 cases measured by PC-MRI at six axial locations along the spine. Error bars correspond to ± 1 standard deviation of flow rates obtained for all 16 NHPs. Note: negative, or peak systolic, CSF flow is in the caudal direction.

*Q*(*t*) of each NHP quantified along the spine had a similar waveform shape, magnitude and axial distribution (**Fig 4**). *Q*(*t*) shape showed a well-defined systolic peak at 100 to 300 ms (negative flow) followed by a diastolic peak that varied based on the heart rate (similar to cardiac blood flow). *Q*_*sys*_ ranged from 0.35–0.87 (ml/s) at the C3-C4 level for all NHPs. *Q*(*t*) at the FM was markedly smaller than at C3-C4. Caudal to C3-C4, *Q*(*t*) had a decreasing trend in magnitude moving down the spine. The CSF flow was found to be nearly zero in all PC-MRI scans before eddy current offset correction. Maximum average CSF flow offset was 13% relative to the arithmetic mean of the absolute CSF flow.

**Fig 5:**
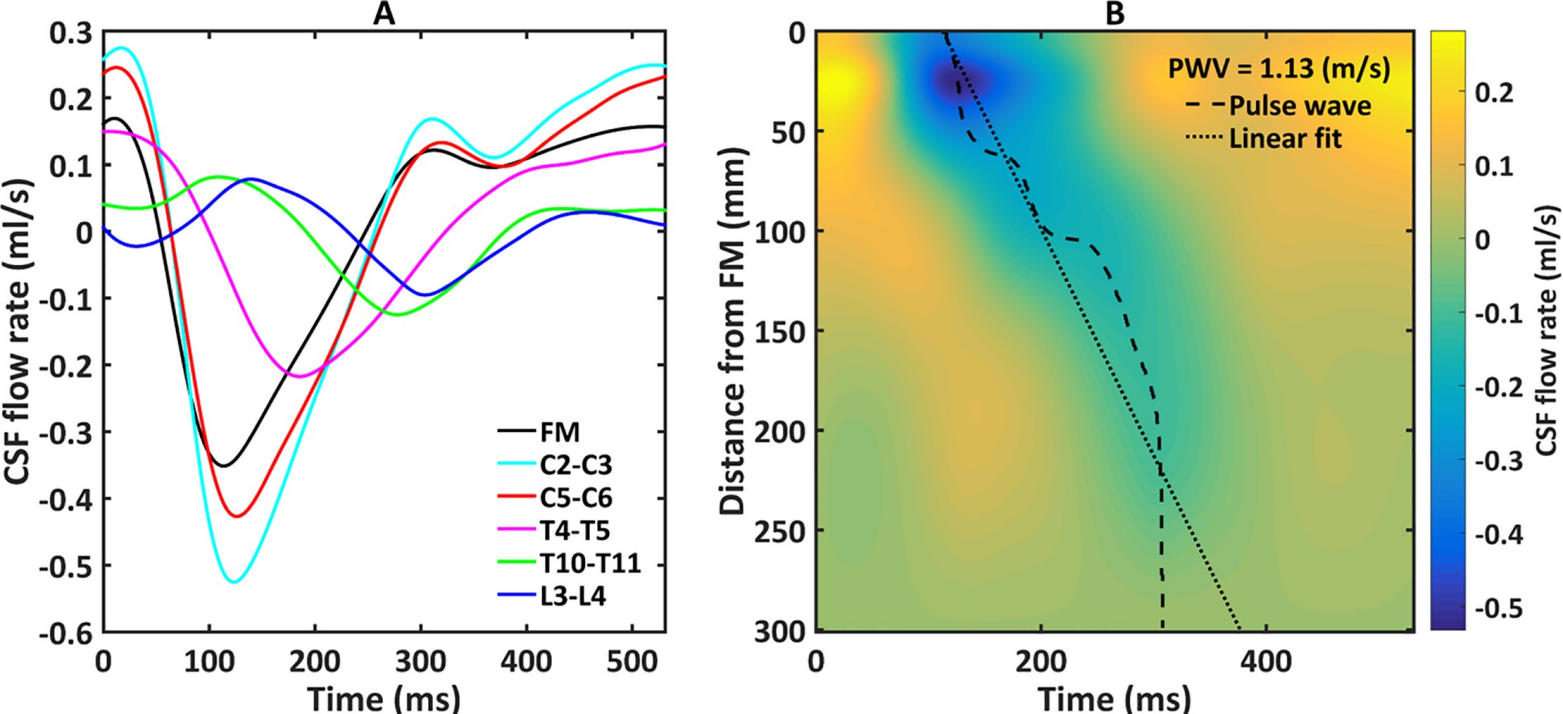
Mean CSF flow waveforms and Spatial-temporal distribution of CSF flow rate. (A) Mean CSF flow waveforms for all 16 cases measured by PC-MRI at six axial locations along the spine. Note: negative, or peak systolic, CSF flow is in the caudal direction. (B) Spatial-temporal distribution of the interpolated CSF flow rate along the spine. Dotted line indicates peak CSF flow rate at each axial level used to compute CSF pulse wave velocity (*PWV*).

Average spatial-temporal *Q*(*t*) distribution across all NHPs showed a relatively smooth decrease in amplitude along the spine and had relatively small, if any, wave reflections from the SAS termination (**Fig 5B**). Spatial temporal *Q*(*t*) distribution showed that maximum CSF flow rate occurred ~25 mm caudal to the FM (**Fig 5B**). *Q*(*t*) shape and magnitude were similar from ~125 mm to the SAS termination.

### Hydrodynamic parameters

**Fig 6:**
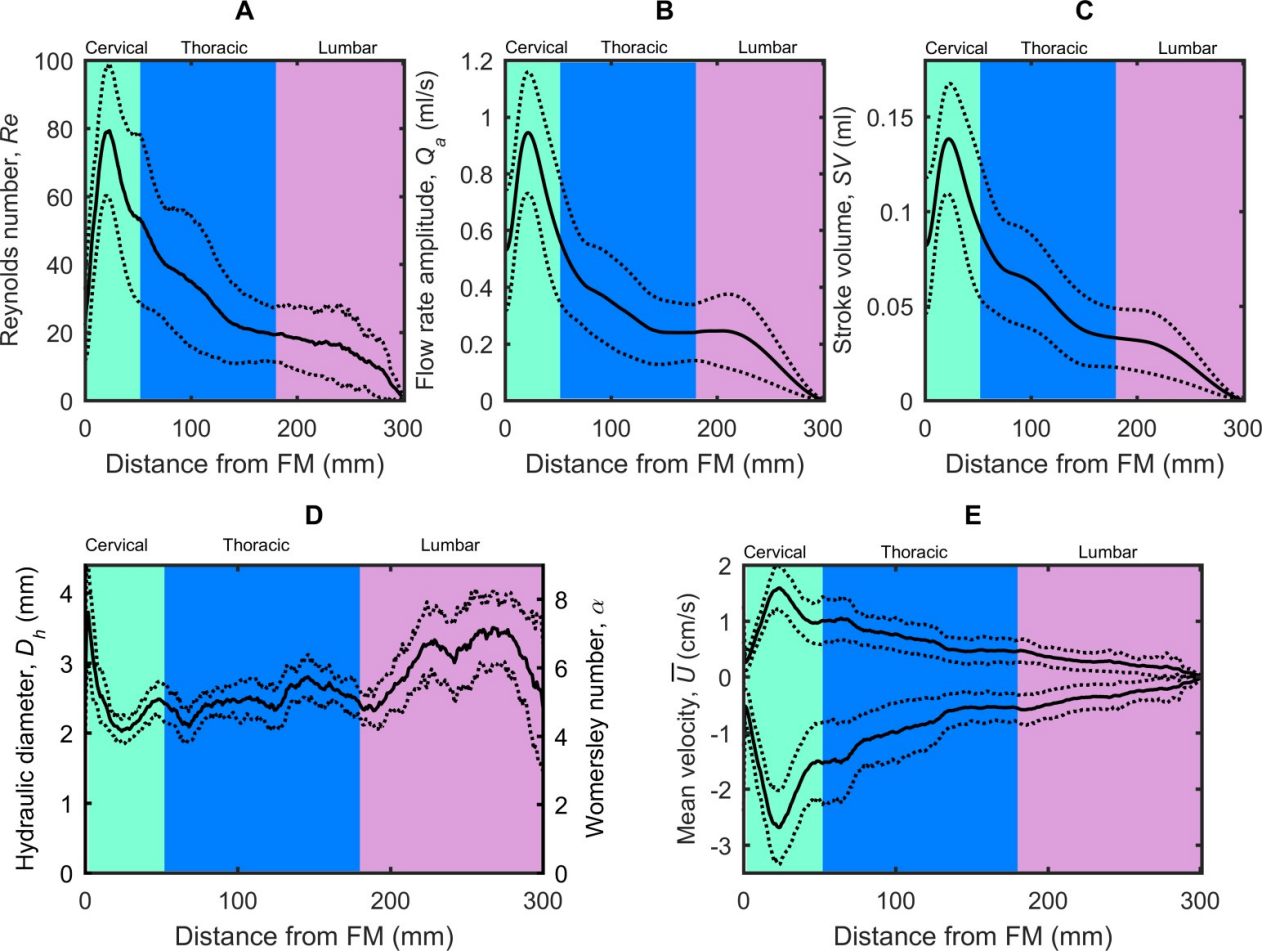
Hydrodynamic parameter distribution computed along the spine for cynomolgus monkeys. (A) Reynolds number, *Re*, (B) Flow rate amplitude, *Q*_*a*_, (C) Stroke Volume, *SV*, (D) left axis, Hydraulic diameter, *D*_*h*_, right axis, Womersley number, *α*, (E) mean peak systolic, ${\bar{U}}_{sys}$, and diastolic, ${\bar{U}}_{dia}$, CSF velocity. Mean value for all 16 NHPs corresponds to the solid line. Dotted lines correspond to ± 1 standard deviation for all 16 NHPs analyzed.

*SV* ranged from ~0 to 0.14 ml along the spine and had the greatest value at the axial location corresponding to C3-C4 (**Fig 6**). Difference in *SV* between NHPs was a maximum of ~ 0.1 ml at the upper cervical spine (C3-C4).

A noticeable phase shift was observed in the *Q*(*t*) along the spine (**Fig 4**). This phase shift is thought to be representative of intrathecal space stiffness or compliance and can be quantified in terms of *PWV*. Based on the time of *Q*_*sys*_ at the FM versus the lumbar spine, *PWV* was estimated to be vary from 0.73 to 3.45 m/s among NHPs with an average value of 1.13 m/s (**Table 3**).

*Re* had a decreasing trend moving caudally along the spine (**Fig 6A**). *Re* varied from 80 in the cervical spine to 0 at the most caudal region, with the maximum value located at C3-C4 level. Local difference in *Re* among the NHPs was a maximum of ~75 and located within the cervical spine. *α* ranged from 4 to 7.7, with a maximum value located near the FM (**Fig 6D**, right axis).

The peak value of the ${\bar{U}}_{dia}$ and ${\bar{U}}_{sys}$ ranged from +1.6 to -2.7 cm/s and occurred at the C3-C4 level (**Fig 6E**). $\bar{U}$ was smaller at the FM compared to C3-C4 for all NHPs. As expected, these alterations in $\bar{U}$ were inversely related to *A*_*sas*_; axial locations with largest *A*_*sas*_ (FM, see **Table 3** and **Fig 3**) demonstrated reduced velocities compared to areas with smaller *A*_*sas*_ and their respective increased velocities.

### Parameter reliability

**Fig 7:**
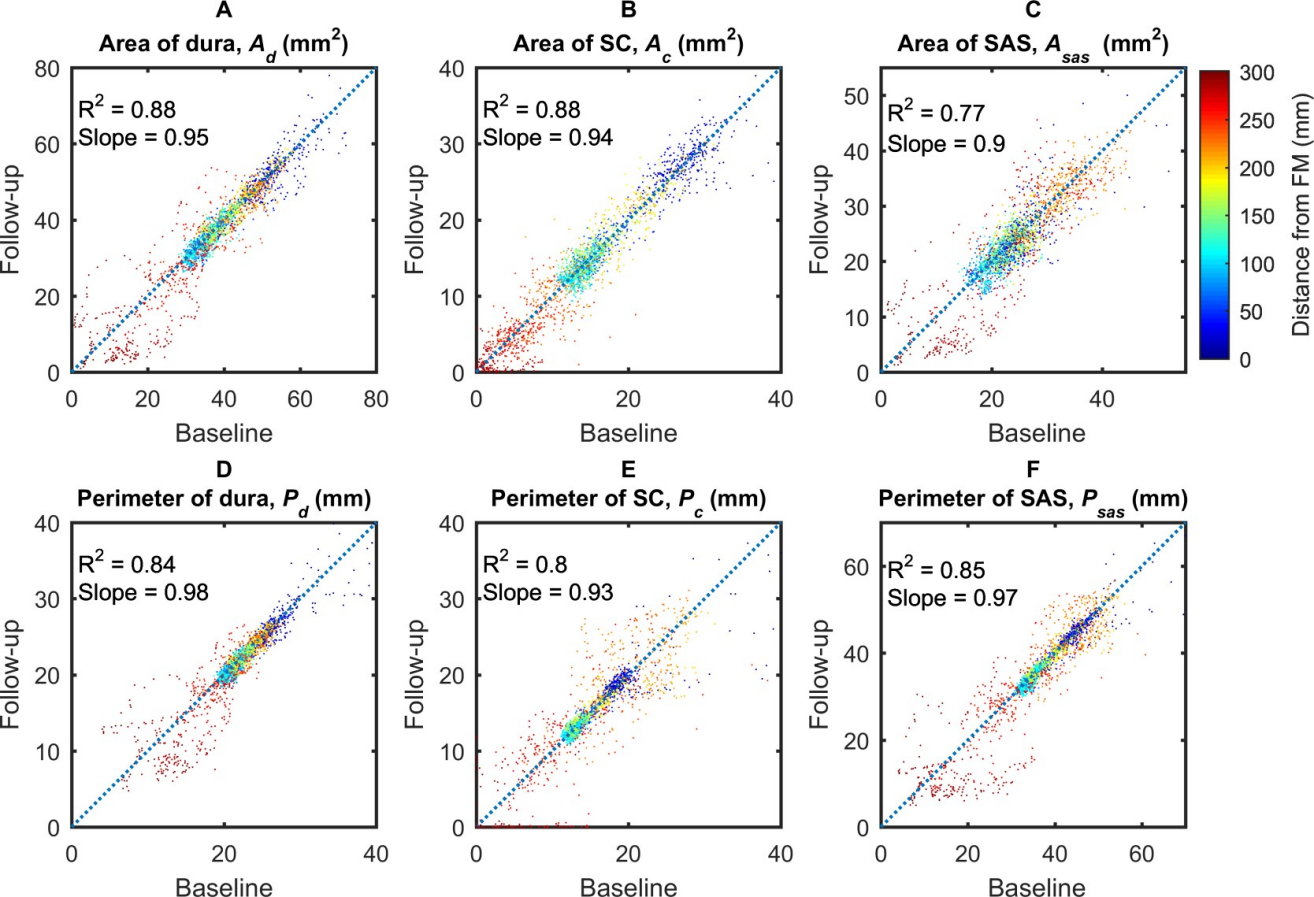
Scatter plots of geometric parameters. (A) Area of dura, (B) Area of spinal cord, (C) Area of subarachnoid space, (D) Perimeter of dura, (E) Perimeter of spinal cord, (F) Perimeter of subarachnoid space. Dot color represents distance from the FM (blue is near the FM and red is near the SAS termination).

**Fig 8:**
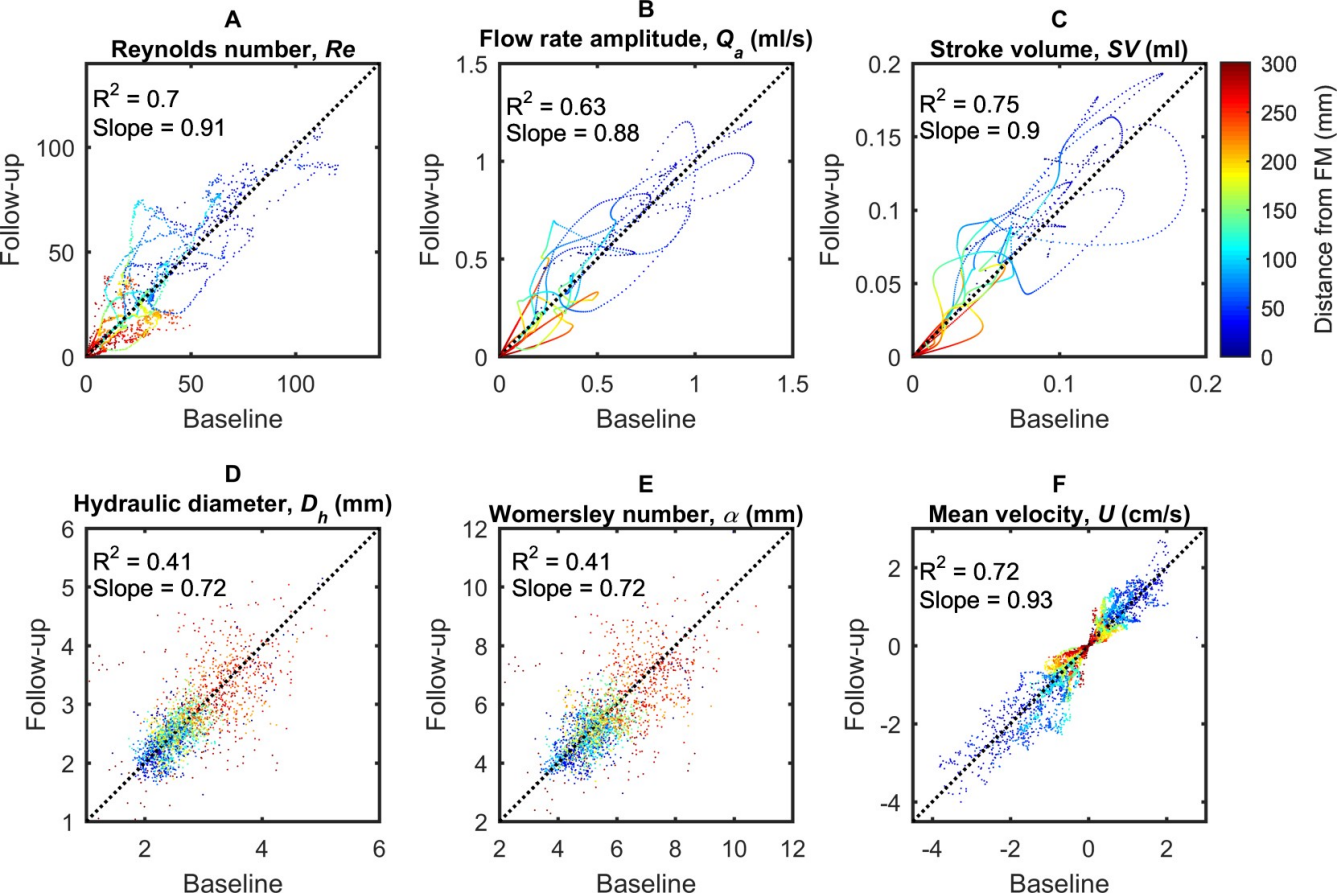
Scatter plots of hydrodynamic parameter distribution computed along the spine. (A) Reynolds number, *Re*, (B) flow rate amplitude, *Q*_*a*_, (C) stroke volume, *SV*, (D) hydraulic diameter, *D*_*h*_, (E) Womersley number, *α*, and, (F) mean peak systolic, ${\bar{U}}_{sys}$, and diastolic, ${\bar{U}}_{dia}$, CSF velocity. Dot color represents distance from the FM (blue is near the FM and red is near the SAS termination).

There was relatively good agreement between the baseline and follow-up MRI scans across all parameters confirming the reproducibility of the method. Differences between geometrics and hydrodynamic parameters obtained from the baseline to the follow-up MRI scan were quantified using regression analysis as shown in **Figs 7** and **8**. The results correspond to all eight NHPs and are plotted for the entire spine model between baseline and follow-up (from FM to the SAS termination).

Strong correlation was observed from the linear regression analysis for *P*_*d*_ (*R*^2^ = 0.84, *slope* = 0.98), *P*_*c*_ (*R*^2^ = 0.80, *slope* = 0.93), and *P*_*sas*_ (*R*^2^ = 0.85, *slope* = 0.97). Correlation was stronger for the *A*_*d*_ (*R*^2^ = 0.88, *slope* = 0.95) and *A*_*c*_ (*R*^2^ = 0.88, *slope* = 0.94), but was slightly weaker in *A*_*sas*_ (*R*^2^ = 0.77, *slope* = 0.90).

The second set of regression plots (**Fig 8**) showed that the value of discrepancy between baseline and follow-up results could be higher for hydrodynamic parameters. There was a relatively weak correlation for *α* and *D*_*h*_ (*R*^2^ = 0.41, *slope* = 0.72). Relative to geometric results, there was additional discrepancy for flow parameters related to $\bar{U}$ (*R*^2^ = 0.72, *slope* = 0.93), *Q*_*a*_ (*R*^2^ = 0.63, *slope* = 0.88), and *SV* (*R*^2^ = 0.75, *slope* = 0.90), though not to the degree as the *α* and *D*_*h*_.

## Discussion

This study presents a method and results for detailed characterization of intrathecal CSF geometry and hydrodynamics in cynomolgus monkeys (*Macaca fascicularis*). Results show that CSF geometry and dynamics can be reliably detected using non-invasive MRI measurements and that results are consistent for cynomolgus monkeys of a similar size and age.

### Nature of CSF dynamics in cynomolgus monkeys

Our results show that CSF moves in a smooth oscillatory manner along the entire spinal axis of cynomolgus NHPs. Chaotic velocity or pressure fluctuations are not expected and transverse CSF velocities (non-streamwise) are likely small compared to axial velocities. CSF dynamics were found to be most active in the cervical spine near the C3-C4 vertebral level with a maximum *Re* of 80 (**Table 3** and **Fig 6**). *Re* was computed to represent the ratio of steady inertial forces to viscous forces and help indicate whether laminar flow (*Re*<2300) was present at each phase contrast slice location (**Fig 6** and **Table 3**). A laminar CSF flow indicates that the flow is smooth with relatively little lateral mixing. This is different from a turbulent flow, where chaotic changes in pressure and velocity occur and can lead to a large increase in lateral mixing. Thus, CSF flow is expected to remain laminar throughout the CSF flow cycle as the *Re* remained sub-critical (*Re*_*critical*_ = 2100) for all NHPs analyzed. However, it is possible that disease states that result in strongly elevated CSF flow velocities (jets) could result in turbulence [34].

Inertial effects are expected to dominate the SAS CSF flow field for normal physiological flow rates, frequencies and CSF fluid properties. *α* varied in the same fashion as *D*_*h*_ with a minimum and maximum value of 4.1 and 7.7 (**Table 3** and **Fig 6**). *α*, was computed to quantify the ratio of unsteady inertial forces to viscous forces that impact the CSF velocity profile shape [35]. For *α*<1, the CSF velocity profiles will be parabolic in shape. *α*>10 will result in relatively flat or plug-like velocity profiles [36]. This means that the CSF velocity profiles will have a plug-like shape throughout the spine. Albeit, flows in an annulus may be less inertial compared to pipe flows of the same *α* [35]. Our previous computational fluid dynamics NHP model indicated a relatively blunt CSF velocity profile in the cervical spine [11]. It is difficult to confirm if the in vivo velocity profiles measured in the current study were in fact blunt shaped (**Fig 2C**) as the MRI resolution was not fine enough to accurately capture the relatively thin boundary layer expected in a blunt flow profile.

### CSF pulse wave velocity along the spine

With each heartbeat, a cardiac-induced CSF pulse wave was found to travel in the cranial-caudal direction (downwards) at a rate of *PWV*~ 1.13 m/s (**Fig 5B**). This wave appeared to be damped along the spinal axis and had relatively little reflection due to the SAS termination. This *PWV* is similar to the study previously reported by our group [11] for one cynomolgus monkey. CSF *PWV* studies have been conducted for humans. Williams obtained simultaneous invasive recordings of ventricular and lumbar CSF pressure in humans during various maneuvers such as coughing and valsalva [37]. From these recordings, a CSF *PWV* can be estimated to range from 8–4 m/s, after coughing. Kalata et al. used high-speed PC-MRI to quantify the CSF velocity wave speed in a small portion of the cervical spine (~20 cm) and found it to be 4.6 ± 1.7 m/s [32]. Another study by Sweetman et al. predicted spinal CSF *PWV* to be ~3 m/s [38]. Martin et al. used a numerical 1-D tube model of the spinal SAS to analyze the effect of dura mechanical properties on spinal CSF flow and pressures and they found CSF *PWV* varied from 2.5 to 13.5 m/s depending on dura elasticity [39]. They also investigated spinal CSF wave phenomena using *in vitro* models and found CSF wave reflections to be present [40]. Similar conclusions have been reported by other groups with different approaches and numerical simulations [41–44]. Results in this study did not show a large degree of CSF wave reflection within the spine (**Fig 5B**).

Arterial *PWV* has been found to have important implications in several vascular diseases [45,46]. Spinal CSF *PWV* could also have implications on perivascular transport in context of syringomyelia [47–49]. Further study is necessary to understand CSF *PWV* in the spine and its relevance CNS physiology in health and disease.

### Geometric and hydrodynamic characterization

To the best of our knowledge, axial variation in spinal SAS geometry in terms of *A*_*c*_, *P*_*c*_, and *D*_*h*_ in a cynomolgus monkey has not been reported in the literature. This may be due, in part, to the relatively long time period (55 minutes total) required to obtain the high-resolution MRI images (375 μm isotopic) used to segment the CSF space in this study. Geometric parameters such as *A*_*d*_, *A*_*c*_, *A*_*sas*_, *P*_*d*_, *P*_*c*_, and *P*_*sas*_ were shown to vary significantly along the spine. Hydrodynamic parameters such as *D*_*h*_, *Re*, *α*, $\bar{U}$, *Q*_*a*_ and *SV* also varied significantly along the spinal canal due to the changes in geometry. CSF flow measurements in the cervical spine by MRI were used to estimate flow values of hydrodynamic parameters. The variation in *A*_*sas*_ is significant ~7 to 75 mm^2^ (see **Fig 3B**), which indicates fluid acceleration may be significant in the spinal cavity near the skull and base of the spine. *D*_*h*_ ranged from ~1.5–4.5 mm in all NHPs analyzed. The axial distribution of SAS geometry in the cynomolgus monkey had a similar trend as that quantified in humans for *A*_*c*_, *P*_*c*_, and *D*_*h*_ [29], albeit approximately ~7.4, 2.3, and 2.4 times smaller, respectively, in magnitude compared to a human [35].

Average *V*_*sas*_ for all NHPs in this study was ~7.41 ml. To our knowledge, *V*_*sas*_ has not been measured in NHPs. However, total NHP CSF volume is typically considered to range from 12–15 mL in CSF dosing studies [50]. Our results indicate that the total NHP CSF volume in these studies is likely to be underestimated by approximately a factor of 2. Similarly, recent studies in humans show that total CSF volume is not 150 mL as reported in the traditional literature [51]. Recent researchers using high-resolution non-invasive MRI-based methods have reported the total CSF volume to be approximately two times larger, ranging from 250–400 mL [52,53]. Detailed MRI investigation of the complete CSF space in terms of its geometry is lacking in the literature.

### Measurement reliability

To help understand parameter reliability, we collected MRI images for 8 NHPs at two time points separated by a two-week time interval. Results showed a relatively strong degree of parameter reliability for all geometric-based parameters (*A*_*d*_, *A*_*c*_, *A*_*sas*_, *P*_*d*_, *P*_*c*_, and *P*_*sas*_ in **Fig 7**) and to a lesser degree for hydrodynamics based parameters (*D*_*h*_, *Re*, *α*, $\bar{U}$, *Q*_*a*_ and *SV* in **Fig 8**). The reason for lower degree of reliability for hydrodynamic parameters is likely because these parameters incorporate input from both flow and geometry, both of which will have associated error and/or natural physiologic variation in NHPs. It should also be noted that we do not expect all parameters to remain identical at the 2-week follow-up time point as CSF flow can be altered due to posture, sedation, and other factors that were not specifically controlled to be identical across MRI scans in the present study. Nevertheless, the degree of reliability is presented to give a benchmark for how much these parameters can change under normal conditions. A previous study by Martin et al. showed a high degree of inter- and intra-operator reliability for MR-based geometric and hydrodynamic parameters derived from the SAS for a single patient with Chiari malformation and a healthy control subject [54]. 2-week follow-up reliability of these parameters was not considered in that study.

### Limitations and future directions

This study provides quantitative measures and reliability assessment for intrathecal CSF dynamics and geometry in eight NHPs. Further studies should quantify potential variance of these parameters in a larger study size across NHP species, age, sex, weight, and in disease states. Geometric characterization did not take into account spinal cord nerve root surface area or volume, which may account for ~231 cm^2^ and ~6 ml, respectively within the SAS in humans [52]. It is expected that these structures will alter the SAS surface area results presented in the current study to a large degree. Albeit, the surface area in contact with the spinal cord and dura is likely similar since the junction of spinal cord nerve roots with these structures is relatively small. Also, we do not expect these structures to alter spinal cord and dura surface area to a great degree or total SAS volume.

There are also a few unknowns in relation CSF flow dynamics. First, CSF flow coupling with the cardiovascular cycle is accounted for in the present study. However, CSF flow is also affected by respiration [55], which was not considered in this study using cardiac-gated PC-MRI measurements. Future studies could investigate the relatively contribution of respiration and cardiovascular pulsations to CSF flow dynamics along the spinal axis. Finally, CSF flow was measured at six axial locations and interpolated to generate a smooth distribution along the spine. The ideal study would minimize or eliminate interpolation as much as possible by adding more axial slice locations. Also, CSF dynamics should be quantified within the intracranial space. However, in the present study, MRI time limitation for each NHP did not allow additional slice measurement locations. The focus of the present study was on the intrathecal space, as this region is most nearby intrathecal therapeutic injection location that can be accessed by lumbar puncture or other relatively minimally invasive procedures. Injection of medications within the ventricular space of the brain or cortical subarachnoid space would also be impacted by nearby CSF dynamics within the ventricles and cisterns of the brain.

## Conclusions

This study presents a detailed geometric and hydrodynamic characterization of intrathecal CSF for eight cynomolgus monkey (*Macaca fascicularis*) with reliability assessed between baseline and a two-week follow-up time point. Results showed laminar CSF flow along the entire spine with maximum CSF flow rate at the C3-C4 vertebral level and peak systolic CSF flow rate and stroke volume at C3-C4. The methods presented demonstrate a reliable method for CSF quantification in NHPs, which may extend in future studies to *Homo sapiens*.

## Supporting information

S1 Table Source data for the axial distribution of subarachnoid space geometric and hydrodynamic parameters and the CSF flow waveforms collected at different vertebral levels. Data for all eight NHPs measured at baseline and follow-up (T1 and T2).
(XLSX)
